# Long-Range, Border-Crossing, Horizontal Axon Radiations Are a Common Feature of Rat Neocortical Regions That Differ in Cytoarchitecture

**DOI:** 10.3389/fnana.2018.00050

**Published:** 2018-06-21

**Authors:** Brett A. Johnson, Ron D. Frostig

**Affiliations:** ^1^Department of Neurobiology and Behavior, University of California, Irvine, Irvine, CA, United States; ^2^Department of Biomedical Engineering, University of California, Irvine, Irvine, CA, United States; ^3^The Center for the Neurobiology of Learning and Memory, University of California, Irvine, Irvine, CA, United States

**Keywords:** horizontal projections, granular cortex, dysgranular cortex, agranular cortex, barrel cortex, motor cortex, multimodal integration, anterograde

## Abstract

Employing wide-field optical imaging techniques supported by electrophysiological recordings, previous studies have demonstrated that stimulation of a spatially restricted area (point) in the sensory periphery results in a large evoked neuronal activity spread in mammalian primary cortices. In rats’ primary cortices, such large evoked spreads extend diffusely in multiple directions, cross cortical cytoarchitectural borders and can trespass into other unimodal sensory areas. These point spreads are supported by a spatially matching, diffuse set of long-range horizontal projections within gray matter that extend in multiple directions and cross borders to interconnect different cortical areas. This horizontal projection system is in addition to well-known area-to-area clustered projections to defined targets through white matter. Could similar two-projection cortical systems also be found in cortical regions that differ in their cytoarchitectural structure? To address this question, an adeno-associated viral vector expressing green fluorescent protein (GFP) was injected as an anterograde tract tracer into granular somatosensory cortex (trunk area), dysgranular cortex (somatosensory dysgranular zone and extrastriate cortex) and agranular motor cortex (MCx). Irrespective of the injection site the same two projection systems were found, and their quantification revealed a close similarity to findings in primary sensory cortices. Following detailed reconstruction, the diffuse horizontal axon radiation was found to possess numerous varicosities and to include short, medium and long axons, the latter extending up to 5.2 mm. These “proof of concept” findings suggest that the similarity of the two projection systems among different cortical areas could potentially constitute a canonical motif of neocortical organization.

## Introduction

Accumulating evidence from functional imaging and electrophysiological studies has demonstrated that stimulating a spatially restricted area (“point” stimulation; e.g., a whisker, a pure tone, or small visual stimulation) in the sensory periphery results in a functional point-spread: a large, roughly symmetrical, diffuse activation, with a radius of several millimeters, in primary sensory cortices, including somatosensory, auditory and visual cortices (reviewed in Frostig et al., 2017). Further, based on restricted anterograde injections, it was demonstrated that a system of short, medium and long-range horizontal projections accompanies and supports such point spreads in the rat’s posteromedial barrel subfield (PMBSF) part of the barrel cortex (Frostig et al., 2008; Johnson and Frostig, 2016) and in other primary cortices such as V1 and A1 (Stehberg et al., 2014). Such evoked activity spreads and their supporting underlying projections ignore cytoarchitectural cortical borders and trespass, sometimes deeply, into other primary cortical areas (reviewed in Frostig et al., 2017). These studies have suggested that at the mesoscopic level, primary sensory cortices contain two anatomical projection systems that include long-range projections: the traditional area-to-area specific projection through white matter, and a roughly symmetrical, diffuse system of horizontal projections through gray matter (Stehberg et al., 2014). The major question that the current project seeks to address is whether the “two-systems” concept is unique to granular primary sensory cortices or whether it might extend to all other known types of neocortical areas including dysgranular and agranular cortices.

To address this question, we have repeated our previous anatomical investigation strategy as employed in the PMBSF, using small AAV virus injections into dysgranular and agranular cortical areas to cause expression of enhanced green fluorescent protein (GFP) under a cytomegalovirus (CMV) promoter (Johnson and Frostig, 2016). Such small injections constitute a “point” injection into cortex and therefore enable us to perform detailed mapping and quantification of the anatomical spread of projections originating from the injection site—i.e., the anatomical point spread. The PMBSF study revealed anatomical point spreads of labeled axons diffusely radiating in all directions for distances >3.5 mm, originating both from supragranular and infragranular injections, with declining density over cortical distance (Johnson and Frostig, 2016). Further, detailed reconstruction of single axons originating from each injection site demonstrated how projections diffusely radiated away from the injection site and across the PMBSF, branched and sometimes crossed into other sensory cortices, as identified by the underlying layer IV cytochrome oxidase staining. Importantly, the study demonstrated that the anatomical point-spread shared many characteristics both with the functional (imaged) point-spread of the same whisker and with detailed mesoscopic mapping of evoked subthreshold electrophysiological recordings. These characteristics include a large and relatively symmetrical spatial extent, ability to cross borders into other cortical areas, and a smooth decline over cortical distance—suggesting together a spatial correspondence between anatomical and functional point-spreads.

Because dysgranular and agranular cortices cannot be directly stimulated by sensory stimulation, the current study focuses only on the anatomical point-spread in dysgranular and agranular areas as compared to granular areas. Our findings serve as a “proof of concept” for a clear similarity among anatomical point spreads in granular, dysgranular and agranular cortices, suggesting a cortical uniformity at the mesoscopic anatomical level and demonstrating that the “two-systems” projections concept has the potential to become a general cortical motif.

## Materials and Methods

### Viral Vector Injections

AAV vectors directing expression of enhanced GFP under a CMV promoter (AAV2/1.CMV.PI.EGFP.WPRE.bGH, PennVector P0101, 2 × 10^13^ GC/ml or AAV1.CMV.PI.EGFP.WPRE.bGH, 2.4 × 10^12^ GC/ml) were from the Penn Vector Core (University of Pennsylvania, School of Medicine Gene Therapy Program). Immediately prior to use, frozen aliquots were thawed and diluted 1:3 in sterile phosphate-buffered saline (PBS: 0.1 M sodium phosphate, 0.9% sodium chloride, pH 7.4) containing 5% glycerol to insure a physiological salt concentration, to conserve the vector and to result in the desired density of labeling. Borosilicate glass capillary micropipettes (1.0-mm outer diameter, 0.25-mm inner diameter) were pulled using a Sutter P-97 pipette puller to produce long narrow shafts that then were cut to give beveled tips and an internal diameter of 7–9 μm (Table 1). Prior to injection, each pipette, loaded with 1–2 μL of virus, was inserted into a stereotactically guided holder that was connected to a Picospritzer II pressure injection system (Parker). After determining the number of pulses needed to eject 100 nL, pressure and duration settings were adjusted to deliver 5–20 nL in 80–100 pulses. In one case (#19), a volume of 80 nL was unintentionally delivered in a single pulse (Table 1).

**Table 1:** Parameters of tracer injections.

Rat	Target^a^	Angle (°)^b^	int. diam. (μm)^c^	Volume (nL)^d^	Survival (days)^e^
18	OC2L/ParP	30	6.7	20	19
19	ParP	30	7.0	80	19
24	S1 PMBSF	45	7.1	5	18
25	S1 PMBSF	45	7.5	20	19
26	S1 PMBSF	45	7.1	20	18
27	OC2L	45	8.4	10	19
30	OC2L	45	7.2	10	18
31	ParP	30	9.2	20	19
33	OC2M	0	9.0	20	13
35	S1 trunk	0	8.4	20	15
36	S1 trunk	0	6.8	20	14
37	MCx	0	6.7	20	13
38	MCx	0	7.0	20	13
39	S1 PMBSF	45	7.0	20	12
41	S1 DZ	45	8.0	20	12
42	S1 DZ	45	8.3	20	11

*^a^OC2L, lateral secondary occipital cortex; ParP, posterior parietal cortex; S1, primary somatosensory cortex; PMBSF, posterior medial barrel subfield; OC2M, medial secondary occipital cortex; MCx, motor cortex; DZ, dysgranular zone. ^b^A value of 0° indicates a vertical orientation of the injection micropipette. ^c^Internal diameter of the micropipette opening. ^d^Volume of viral vector injected. ^e^Time between injection and perfusion*.

Procedures using rats adhered to National Institutes of Health guidelines and were approved by the UC Irvine Institutional Animal Care and Use Committee (IACUC). Sixteen male Sprague-Dawley rats (Charles River Laboratories) between 65 and 74 days of age (mean ± SD: 70.2 ± 4.8) were anesthetized using sodium pentobarbital (i.p., 50 mg/kg), and supplements (i.p., 30 mg/kg) were administered as needed to suppress hindpaw withdrawal and corneal reflexes. Rats also were given injections of ampicillin antibiotic (i.m., 150 mg/kg), 5% dextrose in physiological saline to insure hydration (s.c.), and atropine to control mucous secretions (i.m., 0.05 mg/kg). The left side of the skull was exposed and thinned, a small window was excised over the intended injection site, and the dura was removed. The micropipette was angled as necessary to contact the brain surface orthogonally and was positioned stereotactically to target the area of interest, with small adjustments to avoid blood vessels (Table 1). Case numbers start at 18; missing numbers represent cases from other studies.

Pipette tips were lowered slowly to a depth of 0.3–0.4 mm (cortical layer 2/3). Injections were in discrete pulses delivered at 15-s intervals. After the final pulse, the pipette was left in place for 10 min before being slowly withdrawn. Rats received injections of flunixin meglumine analgesic (s.c., 1.1 mg/kg). The closed wound was covered with topical antibiotic, after which rats recovered from anesthesia, received a second injection of analgesic the following morning, and were housed individually in filter top cages for 11–19 days (mean ± SD: 15.8 ± 3.1, Table 1).

### Histology

Rats deeply anesthetized using sodium pentobarbital (confirmed by absence of hindpaw withdrawal reflexes) were perfused transcardially using PBS followed by 4% paraformaldehyde in 0.1 M sodium phosphate (pH 7.2). Cerebral cortices were flattened to 2-mm thickness between microscope slides and stored at 4°C in 0.1 M sodium phosphate, 30% sucrose (pH 7.4). Transverse slices (40-μm) were prepared using a freezing microtome. The nine most superficial slices were stained using antibodies to GFP to identify axons of infected neurons. Immunohistochemistry involved blocking at room temperature in 5% instant milk, 0.3% Triton X-100 in PBS and incubating overnight at 4°C in rabbit polyclonal anti-GFP antiserum (1:2000 dilution of Invitrogen, A-6455) in 2% milk, 0.3% Triton X-100 in PBS. Visualization used goat anti-rabbit peroxidase (Vectastain Elite ABC kit, Vector Laboratories) followed by a 10-min incubation at room temperature in 0.03% hydrogen peroxide and 0.5 mg/mL diaminobenzidine tetrahydrochloride. Starting at the tenth slice, intermittent slices were stained for cytochrome oxidase activity to localize cortical barrels as well as other primary sensory cortical regions (Wong-Riley and Welt, 1980; Wallace, 1987).

### Microscopy and Image Analysis

Immunostained slices were imaged using an Olympus BX60 microscope, an AxioCam MRm monochromatic camera (Zeiss), and AxioVision Rel.4.6 software (Zeiss). Images of each microscopic field (1388 × 1040 pixels) were saved at two focal depths, and the microscope stage was moved between fields to produce images that overlapped sufficiently to reconstruct the entire slice as a photomontage. After training, individual researchers used computer mice to thoroughly trace images of axon segments detected in these montages; these tracings were made at 1-point thickness in additional layers of the Adobe Illustrator files.

For each brain, images of cytochrome oxidase-stained slices were aligned to one other in separate layers of Adobe Illustrator by matching the locations of blood vessels. Barrels in the somatosensory cortex and boundaries of other sensory cortices (Wong-Riley and Welt, 1980; Wallace, 1987) were traced over these images. Then, montages of immunostained slices together with their traced axons were aligned to the images of the cytochrome oxidase-stained slices and to each other, guided by the locations of blood vessels detected in both types of slices. In this manner, tracings of axons in the nine most superficial immunostained sections were merged to generate overall illustrations of the supragranular axon projection pattern.

Individual long axon segments that appeared to connect with each other between adjacent slices were chosen for detailed reconstruction. To provide a guide for reconstruction, montages of the original 20× images containing these axon segments were assembled in Adobe Photoshop. Then the axon segments were imaged again, typically in dozens of focal planes, through a 100× objective (N.A. = 1.3) to resolve ambiguous crossings, to confirm continuity of the main segment and branches, and to identify bouton-like structures. Using Photoshop, these images of distinct focal planes were loaded into layer stacks, blended for extended focus and overlaid on the 20× montages. As axons approached the injection sites, it generally became more difficult to identify which of the densely packed fragments should be connected across the slices, and these ambiguities usually defined the proximal end of the reconstructed axon. Typically, the distal ends of the reconstructed axons represented terminal fields rich in bouton-like structures. Occasionally, however, the end of a tracing was dictated by the axon’s leaving an immunostained slice and entering either the tenth slice, which was used for cytochrome oxidase staining, or a slice where tissue was missing as a result of a fold or tear.

For quantitative analysis, the stacked, traced slices were rotated to provide a best-fit match to the locations of the posterior whisker barrels in a standard barrel map (Brett-Green et al., 2001). They then were analyzed in MatLab as previously described (Johnson and Frostig, 2016). Briefly, a circle corresponding to a diameter of 7.2 mm was centered on each injection site to define the region of interest in each Illustrator stack. A circle of this size was used in our previous analysis of barrel field injections, where it represented the approximate known extent (~10% of peak response) of the activity spread following individual whisker stimulation (Johnson and Frostig, 2016). Fiber tracings within the circle were clipped and exported as TIFF images (10,800 pixels diameter, grayscale). Tears or large blood vessels within the analysis circle were segmented from the images in ImageJ using the “convert to mask” function, and these images also were exported as TIFF files to be used as a mask during analyses.

The TIFF images were transformed to 500 × 500 item comma-separated value files in which grayscale values were converted to relative scores (saturated, black, areas were given values of 1 and white areas without tracing were given values of 0). After application of the off-section masks, these arrays were resized to 15 pixels in diameter, each cell representing the density of traced axons within a square area measuring 0.48 mm on each side. The resized arrays were averaged across the different section depths for each brain. To better visualize the full range of axonal densities across the analysis region, the arrays were further transformed using the equation: y=x^(log(0.5)/log(x¯)), where *x* represents the original value at each cell of the resized array and $\bar{x}$ represents the mean value across the analysis region for the same slice. Thus, values of 0.5 indicate the mean staining for the analysis region, while values of 0 continue to represent an absence of staining, and values of 1 continue to represent saturated levels of staining such as usually characterized by the injection sites.

## Results

### Injection Sites

**Figure 1:**
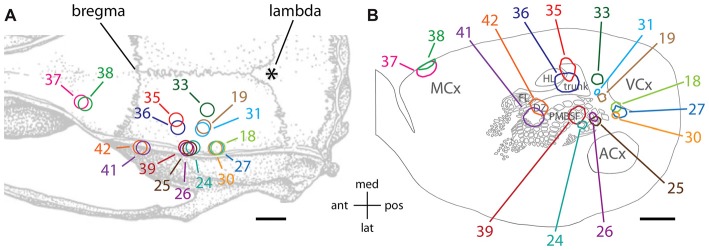
Tracer injections were made into a variety of cortical locations. **(A)** Locations of injections are shown relative to standard skull landmarks. **(B)** The locations of the dark anti-green fluorescent protein (GFP) immunoreactivity at the core of each injection site are shown relative to a single, standard map of the outlines of areas of cytochrome oxidase activity (Brett-Green et al., 2001). For a direct comparison of the injection locations to features of cytochrome oxidase staining detected in slices from the same brains, which can vary from the standard map, please see Figures 2, 3. MCx, motor cortex; FL, forelimb representation in somatosensory cortex; HL, hindlimb representation in somatosensory cortex; trunk, trunk representation in somatosensory cortex; DZ, dysgranular zone of somatosensory cortex; PMBSF, posteromedial barrel subfield of somatosensory cortex; ACx, auditory cortex; VCx, visual cortex; ant, anterior; post, posterior; med, medial; lat, lateral. The scale bars represent 2 mm.

Figure 1A illustrates the locations of the various injection sites in this study relative to skull landmarks, and Figure 1B illustrates the core of the staining from each injection relative to a standard set of features that were evident in different transverse slices of flattened cortex stained for cytochrome oxidase activity. The cores of the injection sites as traced in Figure 1B included stained cell bodies, dendrites and densely packed axon segments, and they likely overestimate the volume of the injections, which typically involved brief pulses to limit the passive extracellular spread of viral particles (Johnson and Frostig, 2016). The staining at the injection site was intense and therefore we were unable to distinguish single neurons even at high magnification.

Two injections (#37 and 38) targeted the forelimb and vibrissa areas of the agranular motor cortex (MCx; Xie et al., 2010) near the border of the primary and secondary subdivisions, M1 and M2 (Paxinos and Watson, 2007). These injection sites were located at the edge of the transverse slices of flattened cortex (Figure 1B). Two injections (#41 and 42) targeted the dysgranular zone of primary somatosensory cortex between the vibrissa and forepaw representations, near the junction of the anterolateral and PMBSFs. Another two injections (#35 and 36) targeted the trunk region of somatosensory cortex (Chapin and Lin, 1984). Four injections (#24, 25, 26, and 39) into the PMBSF were described in a previous article (Johnson and Frostig, 2016). Those injections were analyzed further in the present study in order to make comparisons with the diffuse horizontal axon radiation from PMBSF (Johnson and Frostig, 2016).

In addition, a total of six injections targeted associative, extrastriate cortex located between somatosensory cortex and visual cortex (VCx). This dysgranular region of cortex has been variously partitioned into additional sub-regions based on cytoarchitectonics, physiological responses to light stimuli and/or connectivity (Miller and Vogt, 1984; Coogan and Burkhalter, 1993; Montero, 1993; Rumberger et al., 2001; Palomero-Gallagher and Zilles, 2015; Wilber et al., 2015). Injection #33 was located in a region commonly known as medial secondary occipital cortex (OC2M) or medial secondary visual cortex (V2M), near the border of the medial and lateral subdivisions (Paxinos and Watson, 2007; Palomero-Gallagher and Zilles, 2015). Injections #19 and 31 were in the area of posterior parietal cortex (ParP or PPC: Paxinos and Watson, 2007; Palomero-Gallagher and Zilles, 2015), which receives a dense projection from the PMBSF of somatosensory cortex (Chapin et al., 1987; Koralek et al., 1990; Fabri and Burton, 1991; Aronoff et al., 2010; Johnson and Frostig, 2016). Injections #18, 27, and 30 were located near the rostral part of lateral secondary occipital/visual cortex (OC2L/V2L: Palomero-Gallagher and Zilles, 2015; Paxinos and Watson, 2007).

### Overall Distributions of Supragranular Axon Projections

**Figure 2:**
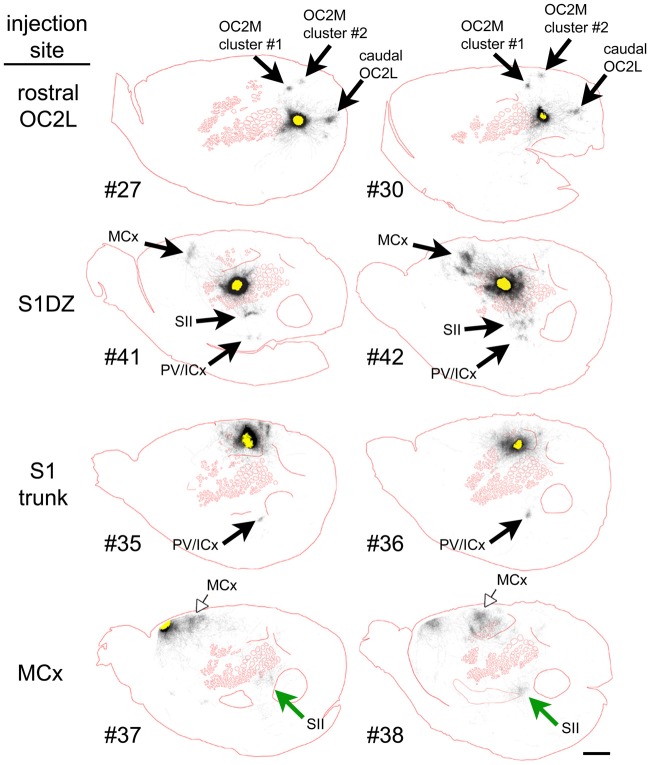
Duplicate anterograde tracer injections into each of four different cytoarchitectonic regions (indicated at far left) revealed a combination of specific projection targets (arrows) and locally dense lateral connections surrounding injection sites, which can be appreciated by tracing the axons using thinner lines. Longer-range horizontal axon segments not clearly related to specific targets are better appreciated by tracing the axons with thicker lines (Figure 3, blue arrowheads). Yellow areas represent the darkest stain at the core of the injection sites, which were invariably surrounded by densely packed axons. The axon segments were traced in each of nine adjacent transverse slices taken starting from the cortical surface, and the tracings were combined across these slices, which were aligned to one another by matching blood vessel patterns. The numbers identify the individual brains injected (see Table 1 and Figure 1). Green and open arrows indicate specific projections that differ in location for the two injections into MCx. Scale bar at bottom right denotes 2 mm. OC2L, lateral secondary occipital cortex; S1DZ, dysgranular zone of primary somatosensory cortex; trunk, trunk representation in primary somatosensory cortex; MCx, motor cortex; OC2M, medial secondary occipital cortex; SII, secondary somatosensory cortex; PV/ICx, parietal ventral/insular cortex. OC2L and OC2M represent cytoarchitectonically defined strips of extrastriate cortex with considerable rostral-caudal extents (Paxinos and Watson, 2007; Palomero-Gallagher and Zilles, 2015). Our injections into the rostral part of OC2L resulted in a clustered projection to a discrete caudal patch that was also located in OC2L, as well as to two discrete patches within the strip corresponding to OC2M, which is consistent with others who have reported discrete subregions of extrastriate cortex (Miller and Vogt, 1984; Coogan and Burkhalter, 1993; McDonald and Mascagni, 1996; Rumberger et al., 2001).

**Figure 3:**
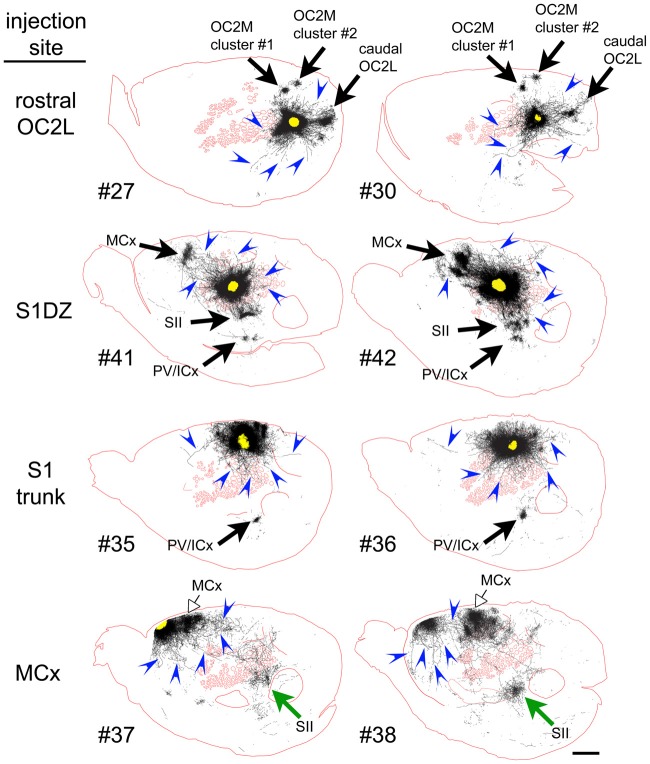
When thicker lines were used to trace the axons labeled after injections of the AAV tracer (Figure 2), longer axons (blue arrowheads) could be seen to radiate in many directions beyond the local network surrounding the injection sites in all regions of cortex that we tested. The numbers identify the individual brains injected (see Table 1 and Figure 1). Green and open arrows indicate specific projections that differ in location for the two injections into MCx. Scale bar at bottom right denotes 2 mm. OC2L, lateral secondary occipital cortex; S1DZ, dysgranular zone of primary somatosensory cortex; trunk, trunk representation in primary somatosensory cortex; MCx, motor cortex; OC2M, medial secondary occipital cortex; SII, secondary somatosensory cortex; PV/ICx, parietal ventral/insular cortex.

To qualitatively assess supragranular axon projection patterns, duplicate injections were made into four cortical areas, namely, the rostral part of the secondary occipital cortex (OC2L), the dysgranular zone of primary somatosensory cortex (S1DZ), the trunk region of primary somatosensory cortex (S1 trunk), and the rostral pole of MCx, followed by a thorough tracing of the axon segments present in the nine most superficial 40-micron slices through the flattened cortices. The results are shown in Figures 2, 3. We have used two different thicknesses of lines for each set of tracings to illustrate different aspects of the projection pattern. In Figure 2, we use thinner lines to emphasize the relative densities of axons around the injection site and in specific projection zones, whereas in Figure 3 we use thicker lines to make evident the more sparsely distributed axon segments that comprise the diffusely radiating horizontal projection pattern.

In general, all of the patterns were characterized by the presence of clustered projections to specific targets (Figures 2, 3, arrows), a dense network of horizontal axons or axon collaterals immediately surrounding the injection sites (Figure 2), and a more diffusely scattered collection of longer, horizontally oriented axons radiating in many directions unrelated to the specific targets (blue arrowheads in Figure 3). The clustered, specific projections (Figure 2, arrows) were distinct for each cortical area, but were usually consistent between the duplicate injections into the same area, with the exception of the injections into MCx. Injection #38 resulted in a clustered projection to more posterior regions of MCx than were labeled for injection #37 (open arrows), and the location of the caudal and lateral clustered projection to secondary somatosensory cortex (SII) also appeared to be distinct for the two injections (green arrows). The general shapes of the dense horizontal axon networks immediately surrounding the injection sites also appeared to be characteristic of each cortical area (Figure 2).

In all cases, isolated individual axon segments were detected well beyond the local horizontal projection, sometimes reaching distances of several millimeters from the cores of the injection sites (blue arrowheads in Figure 3). Unlike the locally dense network of horizontal axons, these segments clearly crossed cytoarchitectonic borders. Following injections into OC2L, labeled axons were found stretching deeply into primary visual and auditory cortex (ACx). Following injections into S1DZ, labeled, horizontally oriented axons were found throughout the body and whisker representation in primary somatosensory cortex as well as running directly into MCx. Following injections into S1 trunk, individual horizontal axons stretched into visual and MCx and across whisker barrel cortex. Following injections into the rostral pole of MCx, axon segments were found distributed across the rest of the MCx, in some cases reaching all the way to the somatosensory cortex.

### Quantitative Comparison of Axonal Distributions

**Figure 4:**
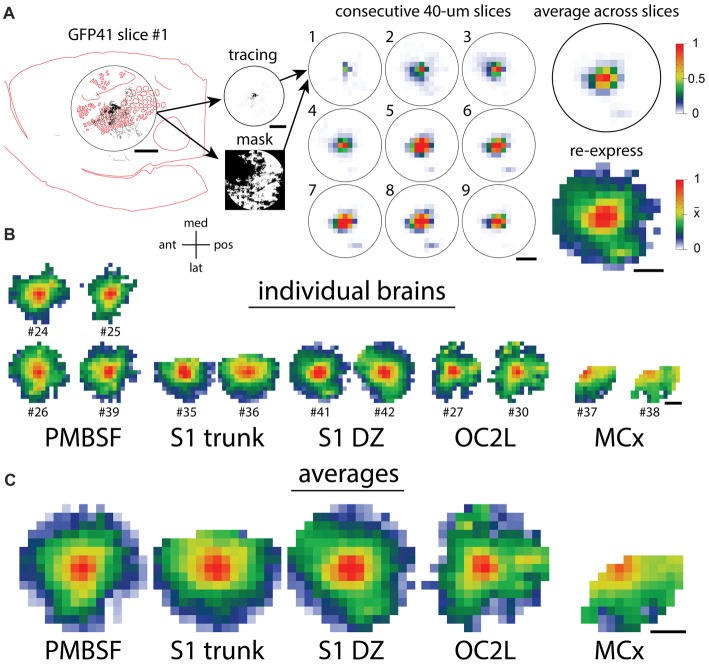
Quantification of axon densities surrounding injections into different cortical areas reveals similarities in the long-range horizontal projection patterns. **(A)** The steps in the quantification procedure (Johnson and Frostig, 2016) are diagrammed. The circular analysis regions are 7.2 mm in diameter, approximately corresponding to the known horizontal spread of activity from a point in the PMBSF upon stimulation of a single whisker. **(B)** Quantitative patterns of axon density that were re-expressed both to correct for different viral labeling intensities and to increase the visibility of low, but non-zero densities show a good agreement between the replicated injections into the same cortical area. **(C)** Quantitative density patterns averaged across the replicate injections reveal axons spreading in all directions throughout each region. Scale bars denote 2 mm. Orientation cross-hairs: med, medial; pos, posterior; lat, lateral; ant, anterior.

To compare the distribution of horizontal axons between different cortical injection sites, we used the method that we applied in a previous analysis (Johnson and Frostig, 2016), and the various steps are diagrammed in Figure 4A. A 7.2-mm diameter circular region of interest was centered on each injection site, and images of tracings from individual slices in this area were converted into smaller arrays. Masks were created for each analysis to exclude regions of missing data due to folds, tears, the presence of blood vessels, or areas of the circular analysis region that were located off the section. The arrays then were averaged across the nine most superficial slices of each brain. These average arrays were visualized after being re-expressed using a non-linear scheme in which a value of 1 represented saturated staining such as occurred at the core of an injection site, a value of 0 represented no staining, and a value of 0.5 represented the mean density value across the analysis area. This re-expression corrected for different staining intensities across different brains and allowed the visualization of axon densities ranging from the highly dense injection sites to the sparsely innervated areas several millimeters away. We applied this analysis to the eight brains shown in Figures 2, 3 as well as to four brains that received injections into the PMBSF in our previous study (Johnson and Frostig, 2016). Figure 4B shows the results for individual brains, and Figure 4C shows the results of averaging the data for the brains that received injections into the same cortical area (Figure 1).

As is evident in Figure 4C, for every injection site, axons extended to the edge of the analysis circle in almost all directions, thereby reaching distances similar to or exceeding those established for the PMBSF. Indeed, the fall-off in axon density with distance in many directions was so similar across the different injection sites that it would be difficult to tell which site was which if one only had a small sector of the circle to evaluate. Some differences in the projection pattern are nevertheless detectable in the transformed data, including the patchy projections to small extrastriate targets following injections into OC2L.

### Reconstruction of Individual Long Horizontal Axon Segments

We previously found that many of the long, horizontal axons radiating from injection sites in PMBSF were confined to supragranular layers of the gray matter throughout the majority (presumably the entirety) of their course, and that many of them also branched several times and possessed numerous bouton-like structures along their way (Johnson and Frostig, 2015, 2016). To determine if this also was the case for injections into other cortical regions, we selected individual axons for re-imaging at higher magnification (100×). Axons that appeared to be moving from one slice to another from near the injection site to their termini were photographed in numerous focal planes, which then were merged and stitched together. Fragments were joined across adjacent slices using blood vessels and other common features for alignment.

**Figure 5:**
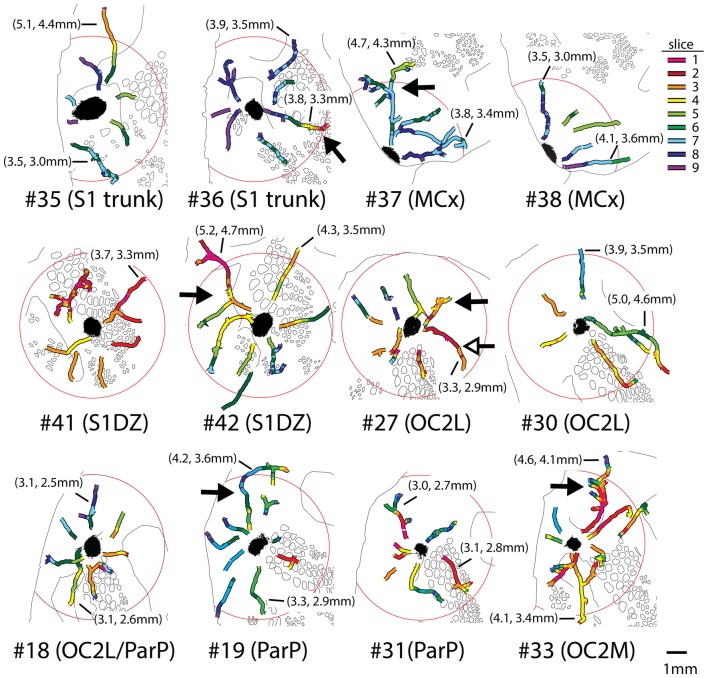
Long axons present in the superficial layers of cortical gray matter were successfully reconstructed from images of segments detected in adjacent 40-μm slices, using blood vessels and other landmarks for alignment between slices. Different colors represent different slices as indicated in the legend at upper right; slice 1 being the most superficial. Circles have radii of 3.6 mm and are centered on the injection sites. Distal ends of tracings usually represented terminal fields, but in some cases marked where the axons either: (1) entered slices in which the tissue was unavailable due to folds or tears; or (2) passed so closely to another axon that it became ambiguous which segment to continue tracing. Proximal endings usually represented ambiguous crossings with other axons in dense regions near injection sites, or, more rarely, where an axon emerged from a region missing from a slice due to a tear or from the tenth slice, which was stained for cytochrome oxidase activity. Outlines of features detected in deeper cytochrome oxidase-stained slices from the same brain are shown in black. Solid arrows denote a selection of axons that branched several times in their course across cortex; details of these axons showing complex branching, varicosities, and bouton-like structures are provided in Figure 6. Photomontages documenting the entire reconstruction of the long axons indicated with solid arrows are also included as supplementary material (Supplementary Figures S1–S6), except for OC2L #27, in which case the reconstruction of the axon indicated by the open arrow is shown (Supplementary Figure S4). Lengths of some of the longer axons reconstructed for each brain are indicated in parentheses; the first value is the distance from the distal end of the axon to the center of the injection site (not the continuous length along the axon, which in most cases would be considerably longer), and the second value is the distance of the distal end from the nearest edge of the dark staining that immediately surrounds the injection site.

Axons were reconstructed for each of the eight injections shown in Figures 2, 3 as well as for four additional injections in medial portions of extrastriate cortex (injections #18, 19, 31 and 33 in Figure 1). For each injection, we were able to establish the presence of continuous axons running in many directions through supragranular gray matter to reach distances of up to 5.2 mm from the centers of the injection sites (Figure 5). Images of entire reconstructed axons (one from each injection site) are shown in Supplementary Figures S1–S6. These axons represent only a subset of the axons that were present at considerable distances from each of the injection sites; when we re-imaged the slices at higher magnification for this analysis, there appeared to be a larger number of axons than were traced from 20× images in Figure 3. Indeed, we abandoned the reconstruction of several axons due to ambiguous crossings with other axon segments. We cannot rule out the possibility that these additional, finer axons that were filtered out by the lower resolution imaging might have affected the overall projection pattern, but we did not perceive any systematic differences in the overall locations or densities of these finer fibers as compared to the traced ones.

**Figure 6:**
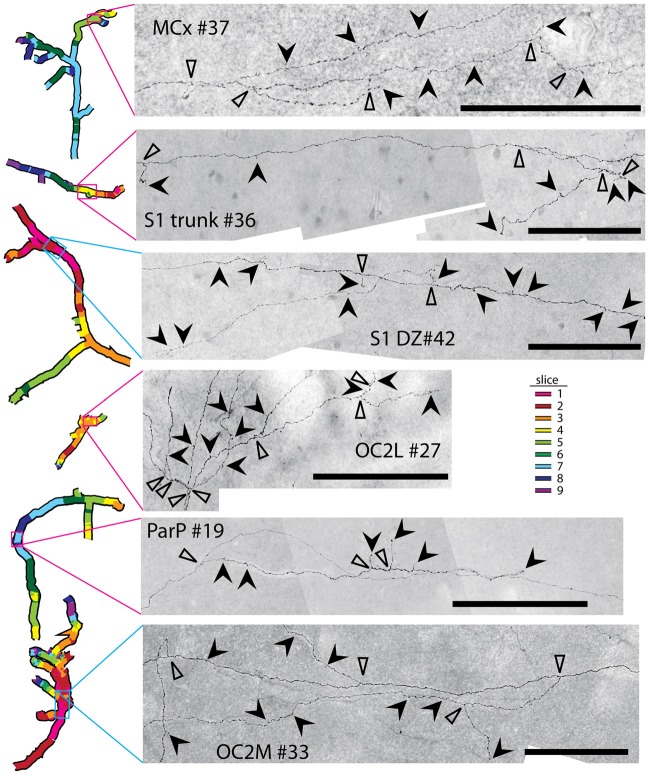
Many axons possessed numerous complex branching patterns, varicosities and bouton-like structures along their course through the gray matter. Details of a small portion of a selected axon from each injection site (see Figure 4 for the location of each axon) are shown. Branch points are indicated by open arrowheads. Bouton-like structures and terminals are indicated by solid arrowheads. Scale bars represent 100 μm. These axon segments also contain intermittent swellings that may represent varicosities supporting additional synaptic contacts.

Some of the reconstructed axons branched on their course through the gray matter (e.g., the axons indicated by solid arrows in Figure 5), whereas others did not branch at all. Both branched and unbranched axons were observed for injections into each cortical region. The low magnification and thickness of lines used in the tracings for Figure 5 in many cases obscured the complexity of the branching patterns. More highly magnified details of some of these axons (indicated by solid arrows in Figure 5) are shown in Figure 6, where instances of branching are more evident (open arrowheads). Many axons also had small processes and thickenings reminiscent of boutons en passage throughout most of their course (Figure 6, solid arrowheads), suggesting that they probably form synapses with many other neurons throughout a large volume of cortex.

### Infragranular Horizontal Axon Radiations

In addition to labeling horizontal axons in supragranular layers, supragranular injections into every cortical area also labeled horizontally oriented axons in infragranular layers. Supplementary Figure S7 illustrates an example in which prominently stained axon segments were traced in five infragranular slices following an injection into layer 2/3 of extrastriate cortex. Stained axon segments extended in most directions from the injection site to reach lengths exceeding 3.6 mm (the radius of the bounding circle in Supplementary Figure S7), although in this particular case, the axons seem to have largely avoided ACx. Diffuse projection patterns were also observed for the other cortical areas into which we injected tracer.

## Discussion

### Summary

The main goal of the present study was to employ a consistent anterograde tracer strategy across cortical regions that differ in cytoarchitecture to determine whether the model of two projection systems, one involving specific, area-to-area clustered axonal projections through white matter to defined targets, and the other involving diffusely radiating, border-crossing, horizontal projections within gray matter, is similar to what we had previously described for barrel cortex (Frostig et al., 2008; Stehberg et al., 2014; Johnson and Frostig, 2016) and other primary sensory cortices (Stehberg et al., 2014). We indeed found evidence consistent with this model as it pertains to both projection systems.

The cortical areas in the current study represent a variety of cytoarchitectonic structures, including granular, dysgranular and agranular regions. Despite this variety, reconstruction of diffuse system axons that were detected in the supragranular layers suggested that they remained within gray matter for the majority, if not the entirety of their course, as was the case for the PMBSF projection (we cannot rule out the possibility that some of the axon fragments that were not reconstructed may have traveled for some distance in other layers). The axons in the diffuse system branched sporadically and often were decorated with varicosities and bouton-like structures suggesting frequent synaptic contacts, although the latter can only be confirmed by employing electron microscopy. In our prior study of the PMBSF, we documented the presence of a parallel axonal radiation in layer 5 following supragranular injections of AAV vector, as well as the presence of axonal radiations in all layers following injections of AAV vector into layer 5 (Johnson and Frostig, 2016). In the present study, we also observed long horizontal axon segments in slices from layer 5 following all supragranular injections into the different cortical regions, consistent with the previous study. In addition to the diffuse, long horizontal projections, injections into each cortical area resulted in a characteristic set of area-to-area clustered specific projections, most of which were known from prior studies. Dense fiber projections to these dense patches were not seen in either the supragranular layers analyzed here or in the infragranular gray matter layers that we also inspected (see Supplementary Figure S7 for an example), from which we infer that the axons likely coursed through white matter to these targets. This class of projections also was noted in our prior study using tracer injections into primary visual and auditory cortices (Stehberg et al., 2014). Together, based on these findings, we suggest that the model of two projection systems could constitute a general motif of cortical connectivity. We cannot exclude the possibility that the two systems are coordinated in their activation; it remains possible, for example, that the same neurons giving rise to the patchy projections send axon collaterals that participate in the more diffuse radiating projection.

### Technical Considerations

In this study, we have interpreted all labeled axons as originating from neurons at the injection site. We cannot, however, distinguish between supragranular neurons and infragranular or pyramidal neurons with dendritic arborizations in the supragranular layers. Furthermore, AAV is known to result in retrograde labeling to some extent (Burger et al., 2004; Cearley and Wolfe, 2007; Taymans et al., 2007; Castle et al., 2014; Wang et al., 2014), and we saw evidence for labeled cell bodies at considerable distances from all of our injections (see Supplemental Figure S8 for an example). Therefore, we cannot rule out the possibility that some of the labeled axons originate from neurons that had taken up the virus at their axon terminals. A systematic comparison of AAV and biotinylated dextran amine as tract tracers concluded that such retrograde labeling was not as significant for AAV, and that AAV also labeled fewer fibers of passage (Wang et al., 2014). Most of the axons that were reconstructed from higher power images in the present study were found to possess terminal fields at their distal extremities, a direct indication that these axons did not originate from retrogradely labeled neurons that simply projected in the opposite direction. In our prior study, we found a similar horizontal axon radiation from barrel cortex following injections of an AAV vector expressing GFP by way of a calcium/calmodulin protein kinase II alpha promoter, which should label only cortical pyramidal neurons; therefore, the labeled neurons in those injections should not have originated subcortically (Johnson and Frostig, 2016). Finally, the presence of some horizontally retrogradely labeled cell bodies likely provides additional supporting evidence for our main findings by highlighting the presence of long-range axons that pick up and retrogradely transport the virus from the injection site to those cell bodies located at different distances within gray matter.

### Comparison to Prior Studies of Cortical Connectivity

There have been numerous prior studies on corticocortical connectivity involving injections of anterograde tracers into cortical regions overlapping with the ones we chose for this study. These prior studies understandably emphasized previously unknown targets receiving dense projections, or they were testing for specific projections based on known physiological phenomena. The clustered, specific area-to-area projection targets that are evident in our study are in close agreement with those reports. Figures from these prior studies often also contain evidence for scattered, widespread horizontal projections through gray matter, but these axons were rarely discussed in the articles, let alone being described in qualitative or quantitative terms or being compared to other cortical areas.

Prior injections into cortical sites similar to our OC2L region (Figure 2), also known as area 18a, resulted in patchy labeling within extrastriate cortex that is consistent with the two patches we observed in OC2M and the posterior patch we detected in OC2L, each patch representing the specific system, suggestive of white matter-based projections to these areas (Miller and Vogt, 1984; Coogan and Burkhalter, 1993; McDonald and Mascagni, 1996; Rumberger et al., 2001). Such injections also resulted in diffuse projections into surrounding gray matter as evident in illustrations showing individual stained coronal slices (Vaudano et al., 1991; Coogan and Burkhalter, 1993; McDonald and Mascagni, 1996). Injections of anterograde tracers into sites similar to our ParP region (Figure 5) also resulted in diffuse labeling several millimeters away, across cytoarchitectonic borders (McDonald and Mascagni, 1996; Smith and Alloway, 2013). Prior anterograde tracer injections into the same area of the S1DZ that we targeted resulted in a very similar pattern of clustered specific projections to MCx, SII and insular or parietal ventral cortex (see Figure 6E of Koralek et al., 1990; and Figure 5, case DZ-a01, of Kim and Lee, 2013) as well as the labeling of isolated axon segments coursing through individual slices of flattened cortex in directions not clearly related to the specific targets (Chapin et al., 1987; Kim and Lee, 2013). Prior injection of tracer into the trunk region of S1 resulted in the labeling of a specific patch similar to the insular or parietal ventral cortex labeling that we observed (Pearson et al., 2001) as well as anterograde fiber labeling within and beyond S1 (see Figure 2H, case R16 of Fabri and Burton, 1991; as well as Pearson et al., 2001). Long, horizontally oriented axon collaterals up to 6 mm in length have been documented for neurons in rat MCx (Keller, 1993; Weiss and Keller, 1994; Huntley, 1997; Kaneko, 2013). The orientation of these axons has been reported to differ in different sub-regions of MCx, being oriented more along the anterior-posterior axis in the vibrissal region and more radially in the forelimb region, but most profusely radiating at the border of these two regions (Weiss and Keller, 1994; Huntley, 1997), where our injections were located. Because our injections also may have straddled the border between primary motor output (medial) and secondary sensory input (lateral) zones of this agranular region (Smith and Alloway, 2013), it is likely that small differences in the location of our duplicate injections can explain the different topography of specific projections to caudal M1 and SII between the two injections.

### Implications of the Evidence for the Presence of the Two-Projection Systems Throughout Different Cortical Areas

If long-range, border-crossing, horizontal axon projections are distributed broadly across rat cortex such as the present data suggests, then one might predict that stimulation of any point in cortex could initiate a spread of activity to surrounding cortical areas that smoothly diminishes with distance along with the decreased density of the projection. In studies using point stimuli that initially activate a limited cortical area, the predicted lateral spread of activity indeed occurs (reviewed by Frostig et al., 2017). In addition, selected examples of direct cortical stimulation include VCx (Orbach and Van Essen, 1993; Huang et al., 2014), limb areas of somatosensory cortex (Austin et al., 2003; Goloshevsky et al., 2011; Hama et al., 2015), insular cortex (Hyde and Li, 2014) and surrounding regions (Fujita et al., 2010), MCx (Capaday et al., 2011), and a variety of other cortical locations (Lim et al., 2012). Electrophysiological analyses of the lateral spreads of activity initiated by microstimulation in mouse VCx (Fehervari et al., 2015) and cat MCx (Capaday et al., 2011) are consistent with an involvement of long-range, thin horizontal axons for at least part of this propagation of activity.

Taken together, these results suggest that activity initiated at different points in cortex can spread across large cortical distances by way of diffuse horizontal projections, which encourages extending our concepts of cortical structure-function relationships. While further research is needed, our findings suggest that potentially every point in cortex (here neocortex) is likely a source and recipient of horizontal axonal projections, including long-range horizontal projections, and some of those very long horizontal projections ignore cortical cytoarchitectonic borders. These findings raise the possibility that under the right conditions neuronal activity in cortex could modulate and be modulated in a distance-dependent fashion over a scale of millimeters. It is important to note that we have focused on the spatial extent of long-range projections, but our findings show a mixture of short, medium, long and extra-long horizontal projections, suggesting that the evoked neuronal ensemble that constitutes the point spread could be comprised of a mixture of multiple and single synaptic projections. Indeed, others have hypothesized that a combination of multiple mechanisms, including electronic coupling, volume conduction and synaptic transmission, may also contribute to evoked activity spreads across neuronal assemblies (Badin et al., 2017).

Why should cortex invest a high metabolic price in developing and maintaining such ubiquitous long-range horizontal projection systems? In other words, what is the reason for the ubiquitous presence of functional point spreads and the anatomical spreads that support them? We have suggested that point spreads reflect neuronal ensembles that constitute a fundamental unit (or motif) of cortical structure-function at the mesoscopic level. Such spreads of activity lead to unique emerging properties such as: providing relative spatiotemporal invariance to changes in stimulation amplitude (Jacobs et al., 2015); serving as building blocks of integrative evoked activity following simultaneous stimulation of multiple points in the sensory periphery (e.g., groups of whiskers; Chen-Bee et al., 2012); helping in unambiguously identifying which point spread was stimulated by which whisker (Jacobs and Frostig, 2017); its amplitude and spatial extent modulation by contextual stimulations; its dependence on behavioral states; and its experience-dependent plasticity characteristics (reviewed in Frostig et al., 2017). Based on the current results, we further hypothesize that these characteristics would likely also be revealed in dysgranular and agranular areas of cortex. Indeed, some supporting evidence for this hypothesis was obtained in agranular MCx. Employing voltage sensitive dyes optical imaging, it was demonstrated that stimulation of a single whisker not only activates the somatosensory cortex, but also the same whisker representation in MCx, which then displayed large scale, border-crossing spreads away from that new peak, similar to the one imaged in PMBSF in the same mouse (Ferezou et al., 2007), results that were recently reproduced in the awake mouse (Kyriakatos et al., 2017). Our current findings are congruent with our previous findings in primary sensory areas (Frostig et al., 2008; Stehberg et al., 2014; Johnson and Frostig, 2016). Together, these findings suggest a potential for a surprising gray matter cortical uniformity at the mesoscopic level, further implying a hypothesis that potentially the entire cortical gray matter processes and integrates evoked neuronal activity in a similar fashion, despite different input and output schemes in the different cortical regions. Our data supports the idea that the output of such processing is sent using the specific projection system via white matter to specific “upstream” and “downstream” cortical and subcortical areas unlike the more diffuse, apparently non-specific spread of the horizontal system within gray matter. Further, our finding regarding the ubiquity of border crossing long-range horizontal projections also suggests that under the conditions of mesoscopic level functional imaging or neuronal recordings employed to map the entire point-spread ensemble, the neocortex could be viewed more as a continuum rather than as a parceled entity (Frostig et al., 2008).

The findings presented in this study, together with our previous findings, suggest that the functional and anatomical point-spread view of cortex (Frostig et al., 2017) should garner further attention regarding the mesoscopic level of cortical structure-function relations. More research is needed to further establish and extend these findings and their potential implications. Our findings regarding cortical regularities such as the anatomical point-spreads seem congruent with recent calls to reveal functional and anatomical regularities in cortex as an optimal strategy for reducing cortical complexity. It is hypothesized that such regularities will point the way to find overreaching large-scale structure-function principles in light of imprecise connectomes and incomplete synaptomes (DeFelipe, 2015; DeFelipe et al., 2016).

## Author Contributions

RF and BJ designed the experiments. BJ performed the experiments. BJ and RF wrote the manuscript.

## Conflict of Interest Statement

The authors declare that the research was conducted in the absence of any commercial or financial relationships that could be construed as a potential conflict of interest.
