# Supplementary material for: Long-Range, Border-Crossing, Horizontal Axon Radiations Are a Common Feature of Rat Neocortical Regions That Differ in Cytoarchitecture
Source: Front Neuroanat. 2018 Jun 21;12:50. doi: 10.3389/fnana.2018.00050 (PMC6021490; doi:10.3389/fnana.2018.00050)
Supplement: Supplementary file 5 [file Image_5.pdf]

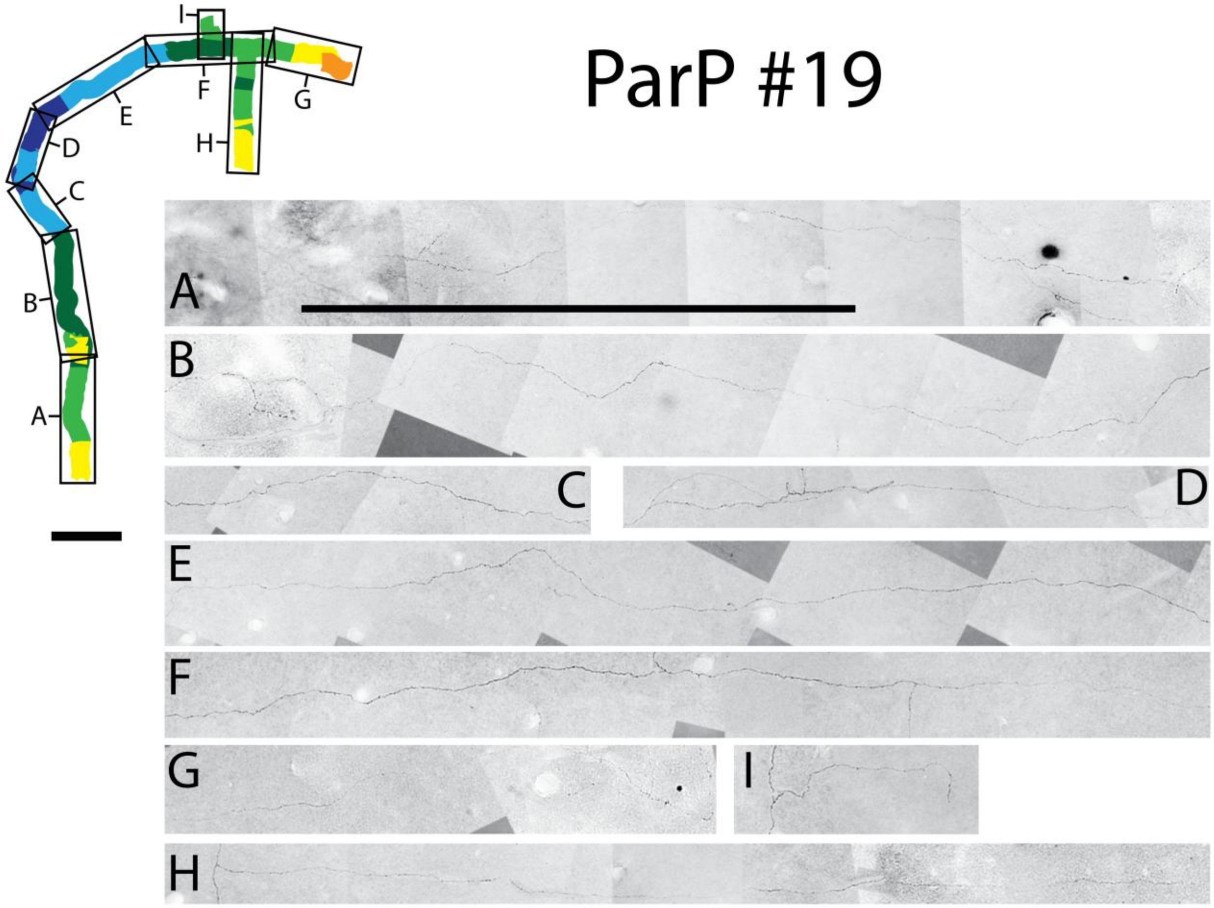

Supplementary Figure S5 | Reconstruction of a long axon from one of the brains injected into posterior parietal cortex (ParP), indicated by a solid arrow in **Figure 5**. (**A-I**) Photomontages corresponding to the regions outlined with rectangles and labeled with the same letters in the inset at upper left. Scale bars represent 500 micrometers. Colors indicate different slices as indicated in the legend to **Figure 5**.
