# Supplementary material for: Long-Range, Border-Crossing, Horizontal Axon Radiations Are a Common Feature of Rat Neocortical Regions That Differ in Cytoarchitecture
Source: Front Neuroanat. 2018 Jun 21;12:50. doi: 10.3389/fnana.2018.00050 (PMC6021490; doi:10.3389/fnana.2018.00050)
Supplement: Supplementary file 7 [file Image_7.PDF]

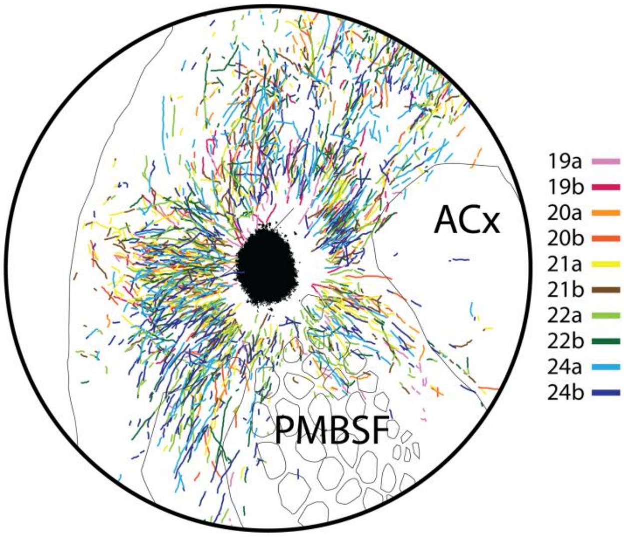

Figure S7: An example of horizontally oriented infragranular axons traced in a brain (GFP 18) that received a supragranular injection of tracer into extrastriate cortex. Axon segments in layer 5 were traced using different colors to indicate the 40-μm section in which they were located as well as their relative depth in that section ("a" indicates a shallower focal plane, whereas "b" indicates a deeper focal plane). The segments were present at similar distances and directions to those found in supragranular layers. Gray lines indicate the edge of the section (left) and borders of features of cytochrome oxidase staining that were detected in layer 4 sections. The outer circle has a radius of 3.6 mm. ACx, auditory cortex; PMBSF, posteromedial barrel subfield.
