# Supplementary material for: Long-Range, Border-Crossing, Horizontal Axon Radiations Are a Common Feature of Rat Neocortical Regions That Differ in Cytoarchitecture
Source: Front Neuroanat. 2018 Jun 21;12:50. doi: 10.3389/fnana.2018.00050 (PMC6021490; doi:10.3389/fnana.2018.00050)
Supplement: Supplementary file 8 [file Image_8.PDF]

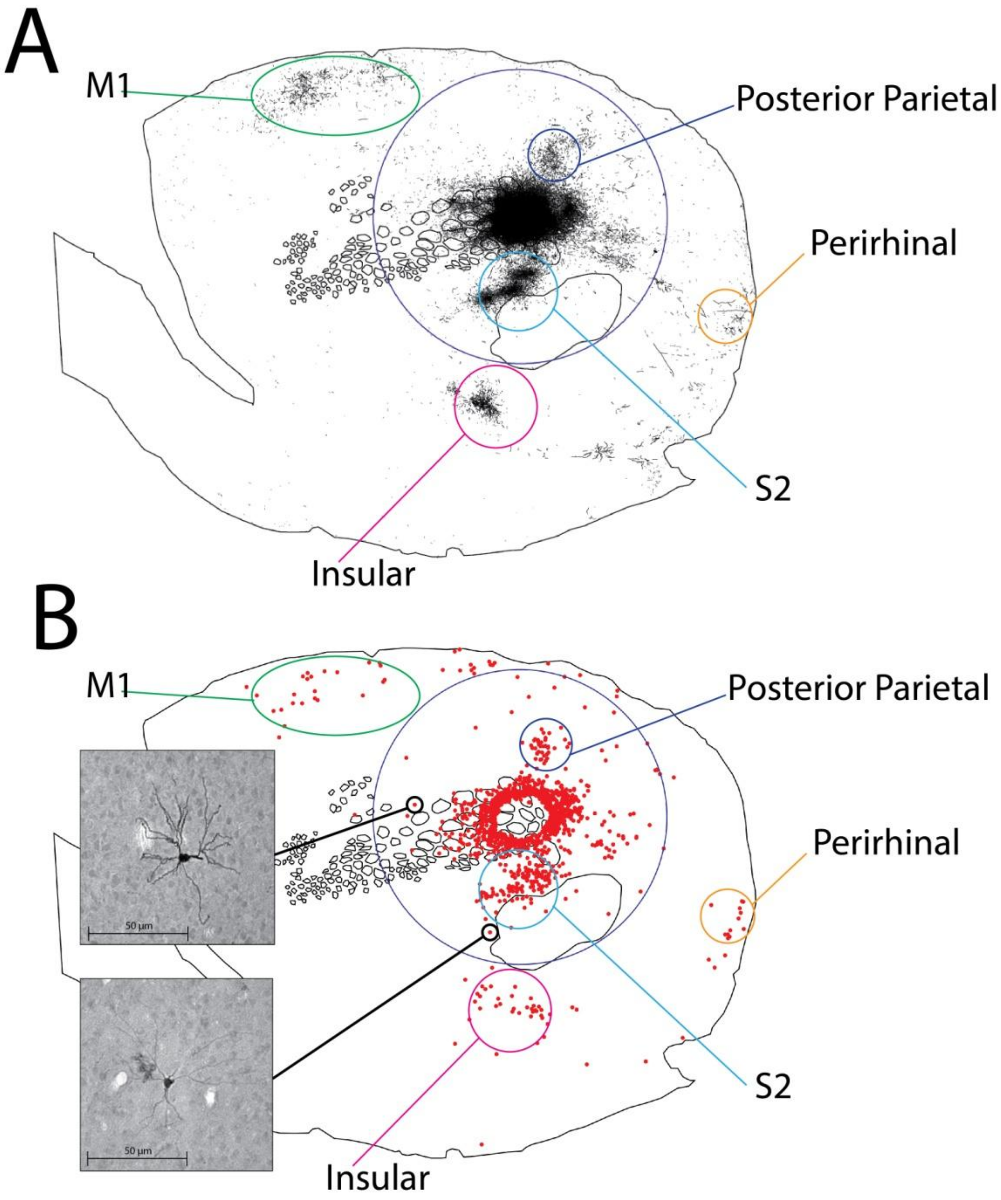

Figure S8: Example of retrograde labeling following an injection centered over the posterior medial barrel subfield. Panel A shows the axon distribution that resulted from the injection, whereas panel B shows the locations of retrogradely labeled neurons. Note the correspondence between the two patterns, the presence of labeled cells in locations known to project to the barrel cortex (colored circles and ellipses), and the scattered labeled cells likely to be relevant to diffuse projections. Photomicrographs of two such cells are shown. M1, primary motor cortex; S2, secondary somatosensory cortex.
